# Spontaneously occurring tumors in different wild-derived strains of hydra

**DOI:** 10.1038/s41598-023-34656-0

**Published:** 2023-05-08

**Authors:** Justine Boutry, Marie Buysse, Sophie Tissot, Chantal Cazevielle, Rodrigo Hamede, Antoine M. Dujon, Beata Ujvari, Mathieu Giraudeau, Alexander Klimovich, Frédéric Thomas, Jácint Tökölyi

**Affiliations:** 1 CREEC/CANECEV (CREES), MIVEGEC, Unité Mixte de Recherches, IRD 224–CNRS 5290, Université de Montpellier, Montpellier, France; 2 MIVEGEC, Unité Mixte de Recherches, IRD 224–CNRS 5290, Université de Montpellier, Montpellier, France; 3 Institut des Neurosciences de Montpellier: Electronic Microscopy Facilities, INSERM U 1298, Université Montpellier, Montpellier, France; 4 Centre for Integrative Ecology, School of Life and Environmental Sciences, Deakin University, Waurn Ponds, VIC Australia; 5 Littoral Environnement et Sociétés (LIENSs), UMR 7266 CNRS-La Rochelle Université, 223 Rue Olympe de Gouges, 17000 La Rochelle, France; 6 Zoological Institute, Christian-Albrechts University, 24118 Kiel, Germany; 7 MTA-DE “Momentum” Ecology, Evolution and Developmental Biology Research Group, Department of Evolutionary Zoology, University of Debrecen, Debrecen, 4032 Hungary

**Keywords:** Cancer models, Germ cell tumours, Developmental biology, Germline development, Pathogens, Zoology, Animal physiology, Ecology, Ecophysiology, Freshwater ecology, Microbial ecology

## Abstract

Hydras are freshwater cnidarians widely used as a biological model to study different questions such as senescence or phenotypic plasticity but also tumoral development. The spontaneous tumors found in these organisms have been so far described in two female lab strains domesticated years ago (*Hydra oligactis* and *Pelmatohydra robusta*) and the extent to which these tumors can be representative of tumors within the diversity of wild hydras is completely unknown. In this study, we examined individuals isolated from recently sampled wild strains of different sex and geographical origin, which have developed outgrowths looking like tumors. These tumefactions have common features with the tumors previously described in lab strains: are composed of an accumulation of abnormal cells, resulting in a similar enlargement of the tissue layers. However, we also found diversity within these new types of tumors. Indeed, not only females, but also males seem prone to form these tumors. Finally, the microbiota associated to these tumors is different from the one involved in the previous lineages exhibiting tumors. We found that tumorous individuals hosted yet undescribed *Chlamydiales* vacuoles. This study brings new insights into the understanding of tumor susceptibility and diversity in brown hydras from different origins.

Tumors are defined as an uncontrolled proliferation of “cheating cells” within an organism^1,2^, which can even spread into surrounding tissues or distant organs (metastasis), in case of cancer. The presence of “cheating cells”, a constitutive challenge associated with the evolution of multicellularity, affects the vast majority of metazoans from all ecosystems^3–5^. The field of cancer research has first developed in the biomedical community, away from evolutionary and ecological sciences. During the mid-seventies, pioneering papers started to transform our understanding of oncogenic processes^6,7^, but it was only during the last decades that evolutionary understanding of malignant processes acquired its recognition^4,8–10^. At the same time, ecologists started to understand the importance of cancer for wildlife and ecosystem functioning^4,11,12^. However, studying cancer in natural populations is challenging: scientists have to face the difficulty to detect them, especially when it concerns tumors that have no external manifestations. In addition, affected individuals may suffer from a higher direct or indirect (e.g., predation, parasitism) mortality rate, and thus disappear rapidly^5,12–14^. These limitations explain why cancer in wildlife is still underestimated and understudied, while laboratory biological models of cancer are common. However, because of the increase of anthropogenic pollution in ecosystems and its association with cancer incidence, this topic became timely and crucial^11^.

Over the past 270 years, the freshwater polyp hydra became a valuable biological model for major advancements in various fields of study such as developmental biology, physiology, sexual reproduction, aging, and animal behavior^15,16^. Domazet-Lošo and colleagues^17^ even extended the potential of this model to address questions in oncology by reporting the first case of naturally occurring tumors in hydras. Two strains developed spontaneous tumors, composed of an accumulation of germline stem cells (referred as GSC hereafter) in the ectoderm. Those tumoral polyps over-expressed 44 genes that are common with genes overexpressed in human tumors^17^. These tumors in hydras can be horizontally propagated to other individuals through experimental grafting, but are also naturally transmitted to the next generation through asexual reproduction (i.e., budding)^17^, a rare characteristic for tumors that brings them closer to the category of transmissible cancers like the facial tumors in Tasmanian devil (*Sarcophilus harrisii*), a veneral transmissible cancer in dogs, or transmissible leukemia in different species of bivalves (see^18^ for more details).

In 2020, Rathje and colleagues^19^ discovered that the development of tumors in one strain of *H. oligactis* (St.-Petersburg^17^) is induced and can persist only with the presence of a bacterial conflict between two bacteria (*Pseudomonas* and the spirochete *Turneriella*), while in the other hydra species there is no difference in the microbiome of healthy and tumorous hydras^19^. *Pseudomonas* are commensal bacteria naturally present in the microbiome of *Hydra oligactis*, while spirochetes have only been reported previously in *Hydra circumcincta* before^20,21^. Only if both bacteria co-occur in the mesoglea of a Hydra polyp, they start expressing a plethora of putative virulence factors. These, in turn, appear to alter morphology and physiology of Hydra cells resulting in tumor formation. Interestingly, no substantial difference in the microbiome composition between healthy and tumorous *P. robusta* polyps has been detected, and the tumorous phenotype could not be eliminated by antibiotic treatment (in contrast to St. Petersburg strain), suggesting an alternative, microbiome-independent mechanism of tumor formation in *P. robusta*^19^.

More recently, we showed the impact of the tumoral phenotype on biotic interactions within ecosystems, through, for instance, an enhancement of hydra predation risks^14^. However, even if these tumorous hydras offer a fascinating opportunity to study the evolutionary ecology of host-tumor relationships, the existence of this phenomenon in natural environments remains to be determined.

Here, we investigated different cases of potential tumorous individuals spontaneously appearing in various strains of *Hydra oligactis*. Those individuals were isolated from wild sampled individuals of various geographical and laboratory origins. We describe and compare their physiological organization, their histological features, and the composition of their bacterial communities. In addition, we compared the characteristics of these outgrowths with the tumors already described in the previous strains in order to identify the commonalities between tumors at the species level or differences within lineages. This study will improve our knowledge of a biological model that is already widely used, thus opening new connections with oncology.

## Results

The hydra strains presented in this study are from individuals collected in different geographical locations (see Table 1), three strains are females (St. Petersburg, Montaud, X11/14) and one is a male (C2/7). The first individuals showing visible swellings among those lineages were observed in the cultures after a period varying between one and four months (see Table 1).

**Table 1:** Collection date, location, sex and tumorous isolation date of the different strains of *Hydra oligactis* used in this comparative study.

Lineage	Sampling location	Date of sampling	Sex	Date of the observation of the first tumorous indvidual
St. Petersburg	58°81′23″N; 29°98′55″E	2007	Female	2007 (one month after^22^)
Montaud	43°44′52″N; 3°59′23″E	April 2021	Female	August 2021
X11/14	47°67′12″N; 20°86′41″E	August–September 2016	Female	December 2016
C2/7	47°67′12″N; 20°86′41″E	August–September 2016	Male	December 2016

### Morphological and histological organization

**Figure 1:**
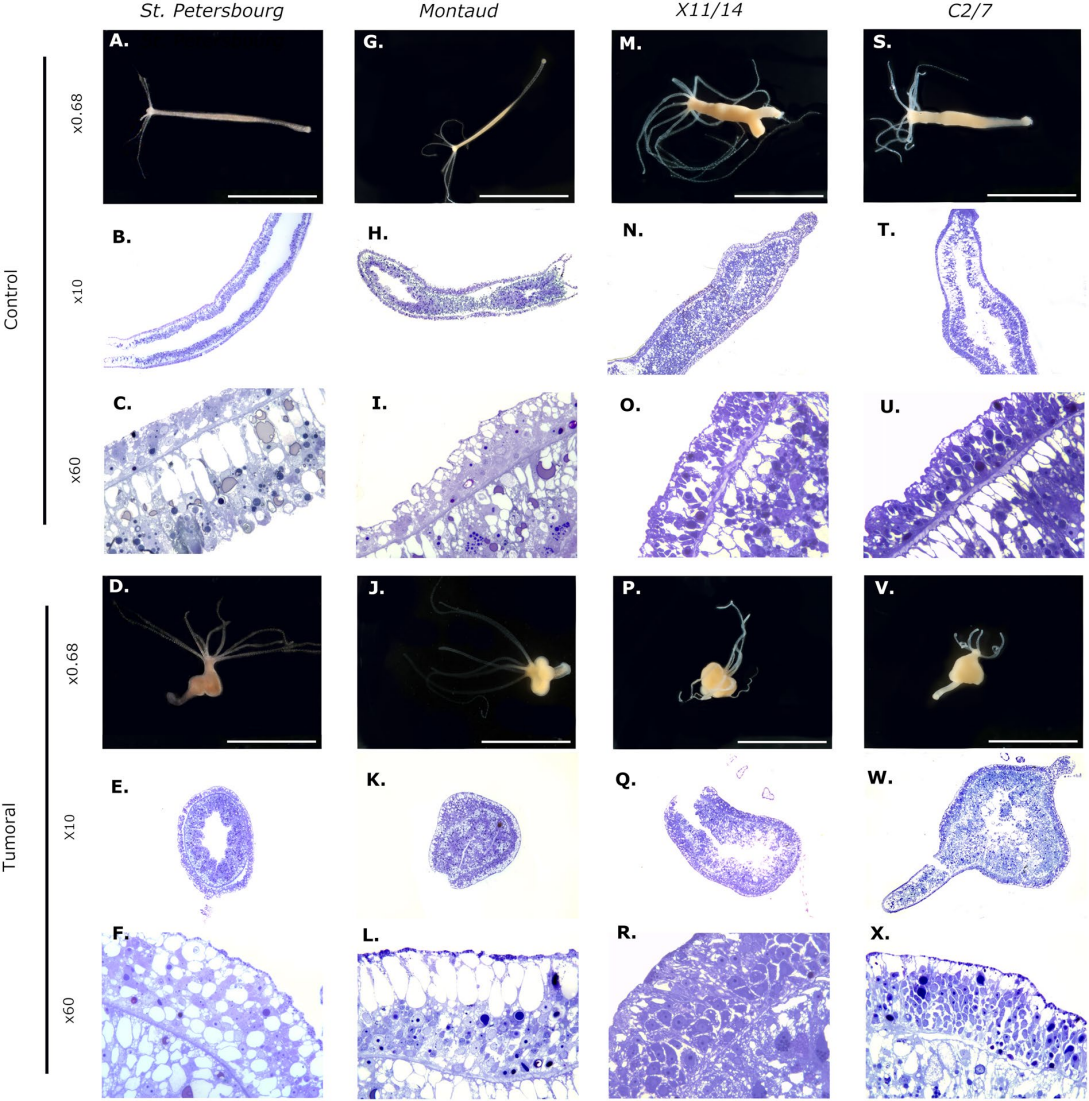
Tumors in three wild-derived strains of *Hydra* have a similar tissue organization to the tumors of St. Petersburg strain. Morphology of normal (**A**, **G**, **M**, **S**) and tumorous (**D**, **J**, **P**, **V**) individuals of *H. oligactis* strains at 0.68x. Histological longitudinal cross-sections through polyp body column at the 10X magnifications of tumorous polyps (**E**, **K**, **Q**, **W**) compared to the control ones (**B**, **H**, **N**, **T**). Pictures of longitudinal cross-sects. (100x) of cells in the ectoderm of the control (**C**, **I**, **O**, **U**) individuals compared to the tumorous ones (**F**, **L**, **R**, **X**).

To verify that the swellings visible on these individuals resulted from the accumulation of abnormal cells and could therefore be qualified as tumors, we performed histological sections to visualize the cellular composition of the different tissues. Results obtained were compared to the St. Petersburg lineage (Fig. 1A–F), that was previously described as able to harbor tumors in the literature^17,19^. Consistent with these studies, tumorous individuals of the reference strain showed an altered phenotype with visible nodules and an increased number of tentacles (Fig. 1A–F) and a significant enlargement of both epithelia (endoderm and ectoderm, Fig. 1E,F) compared to healthy polyps (Fig. 1B,C). The three recently sampled lineages, Montaud (Fig. 1G–L), X11/14 (Fig. 1M–R), and C2/7 (Fig. 1S–X) also show a similar enlargement of the epithelia at the histological level. Remarkably, interstitial stem cells (ISCs) seem to be locally aggregated in the ectoderm of X11/14 tumorous individuals and appear visually enlarged (Fig. 1R), whereas ISCs in C2/7 tumor ectoderm appear smaller, but still in greater density (Fig. 1X; additional histological slides are available in the Supplementary material) compared with healthy polyps. In contrast, the cells of the endoderm, although more numerous, have a homogenous and normal appearance (see Fig. 1 and additional slides in the Supplementary).

**Figure 2:**
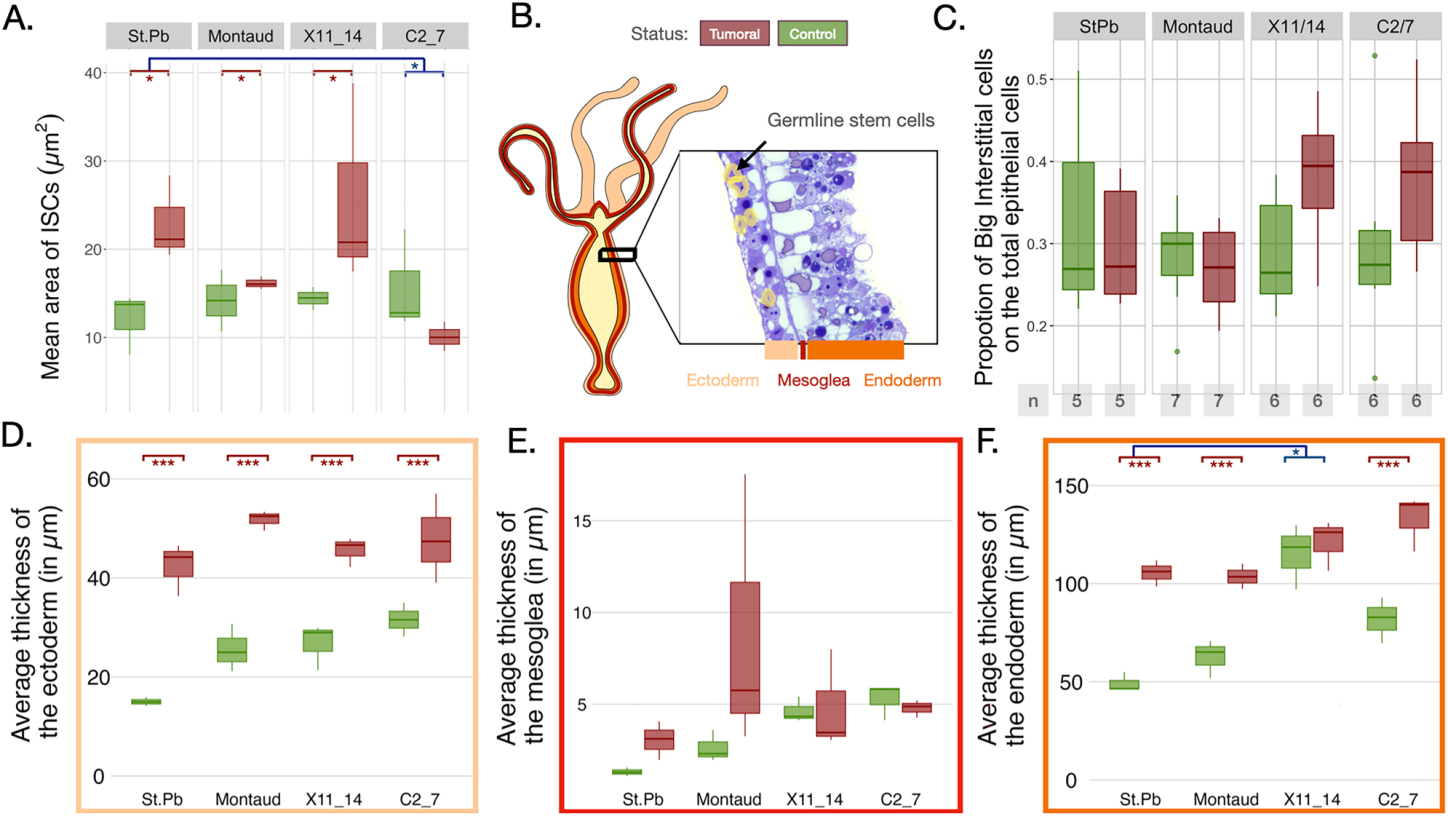
Tumors are associated with modifications of the stem cell traits and tissue thickness in all strains. (**A**) The average area of the interstitial stem cell (ISC), estimated on three ISC of three different slides of a control (in green, on the left) and a tumorous (in red, on the right) individual of each strain (in µm^2^). (**B**) The schematic representation of tissue organization and germline stem cells in hydras. (**C**) Proportion of big interstitial stem cells (ISC) in the ectoderm of control and tumorous individuals of each strain, number of individuals studied is indicated in the boxes below the graph for each strain. The average thickness of (**D**) the ectoderm, (**E**) the mesoglea and the (**F**) endoderm of a tumorous and a control hydra (in µm), measured on three different slides. Significant variations between tumor and healthy individuals are represented by comparison bars with stars according to p values (*p < 0.05, **p < 0.01, ***p < 0.001). Statistical differences solely attributable to inter-strain variation are not represented in the graphs (see in the Supplementary material).

Beyond the appearance of the tissues, we also measured and compared their thickness. The ectoderm thickness of tumorous (i.e., previously qualified as swollen) individuals was widely increased in all strains (Fig. 2B,D; *p*-value = 1.83e−09, see details in Supplementary Table 1), while the mesoglea was not significantly enlarged in the tumorous individuals (Fig. 2B,E, *p*-value = 0.15, see details in Supplementary Table 2). Finally, the average thickness of the endoderm was also increased in all strains in tumorous (i.e., swollen) individuals, except for the X11/14 strain (Fig. 2B,F, *p*-value = 0.005, see details in Supplementary Table 3). However, we suspected here that the axis of this histological section was not central (see in Supplementary material).

We also measured two other features potentially altered in tumor cells; the area of the ISCs (on these same sections) and the number of ISCs (with macerations), to compare if those cells exhibit convergent tumorous phenotypes. The mean area of ISCs shows an interaction with strain (Fig. 2A; *p*-value = 0.05, see details in Supplementary Table 4 such that it increases significantly in tumorous (i.e., swollen) compared to non-tumorous females, while it is the opposite for the male strain C2/7 which are smaller for the tumorous (i.e., swollen) individuals (Fig. 2A, see details in Supplementary Table 4). The macerations of individuals from these four strains did not allow us to see any differences in the proportion of ISCs compared to the total number of epithelial cells in any strains (Fig. 2C, *p*-value = 0.19, see details in Supplementary Table 5, even if a non-significant trend for an increase may exist for the two Hungarian strains, X11/14 and C2/7, looking at the plot.

### Microbiome description

The 16S rDNA metabarcoding analysis used to assess the bacterial microbiome of non-tumorous and tumorous hydras of the four lineages was performed on 70 individuals. The elimination of OTUs with less than 50 reads (after removing contaminating and false-positive OTUs) allows us to reduce our dataset from 3282 to 64 OTUs by losing only 4% of the reads, which led us to 7,892,262 sequences. Of these sequences, 92% of the reads are from 5 major taxons (*Chlamydiales*, *Solilurobacteriales*, *Leptospirales*, *Pseudomonadales* and *Burkholderiales*).

**Figure 3:**
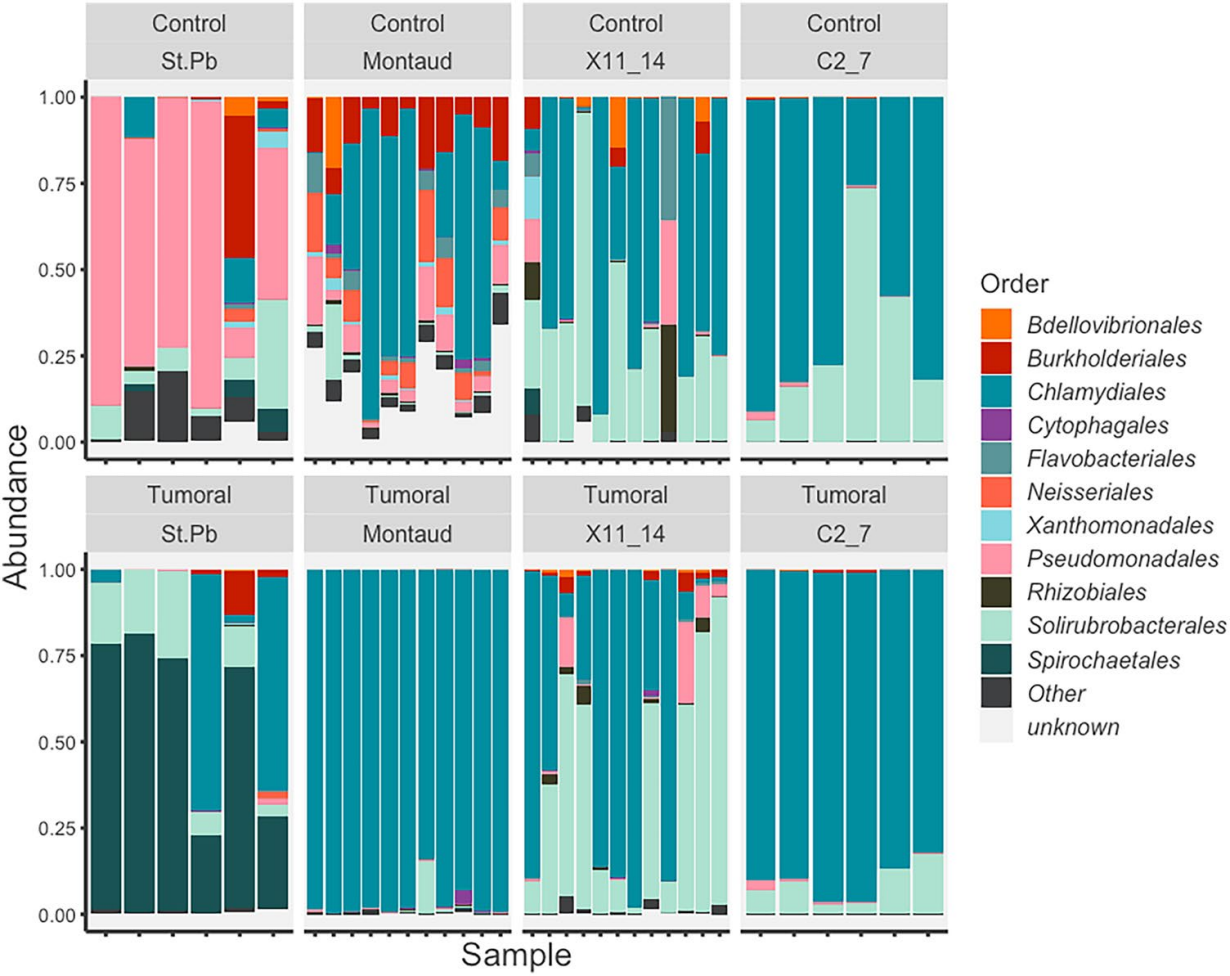
The microbiome differences associated with tumors in the different strains. Relative abundances plots of the microbial composition of healthy (control, above) and tumorous polyps (tumoral, below) were identified by 16S rDNA sequencing and presented the bacteria order level of the 96% most prevalent orders.

Relative abundance values presented in Fig. 3 illustrate the presence and absence of different bacterial genera detected in the control and the tumorous hydras of the four studied strains. First, we detected a majority of *Pseudomonadales* in the control individuals from St Petersburg, while Spirochaetales are the most represented in the tumoral individuals of the same strain. This is consistent with the previous literature, with the exception that we do not detect co-infection in all individuals or in a lesser extent than reported previously^19^. This might be explained either by the too low sensitivity of our analysis compared to the prevalence of *Pseudomonas*, or by a secondary loss of *Pseudomonas* within our culture or even a replacement of the latter within the interaction^23^. Unexpectedly, in the majority of the hydras tested (both healthy and tumorous), we detected the presence of *Chlamydiales* in their microbiota, including St. Petersburg individuals. Considering the amplicon sequences, this *Chlamydiales* is only represented by an identical sequence from yet undescribed strain closely related to a family of environmental *Chlamydiales* previously sampled in activated sludges, anoxic water, and lava caves. Finally, we found that control individuals from Montaud contained a substantial fraction of unidentified bacteria in their microbiome (see Supplementary Fig. 1. for the beta-diversity analysis), this observation would require further investigation given that the method used here is only semi-quantitative. Within strain, tumorous and non-tumorous individuals from Montaud, X11/14 and C2/7 differed much less in microbiota composition than St. Petersburg, although the abundance of *Burkholderiales* non-described bacteria seemed higher in controls compared to tumorous polyps of the Montaud strain.

**Figure 4:**
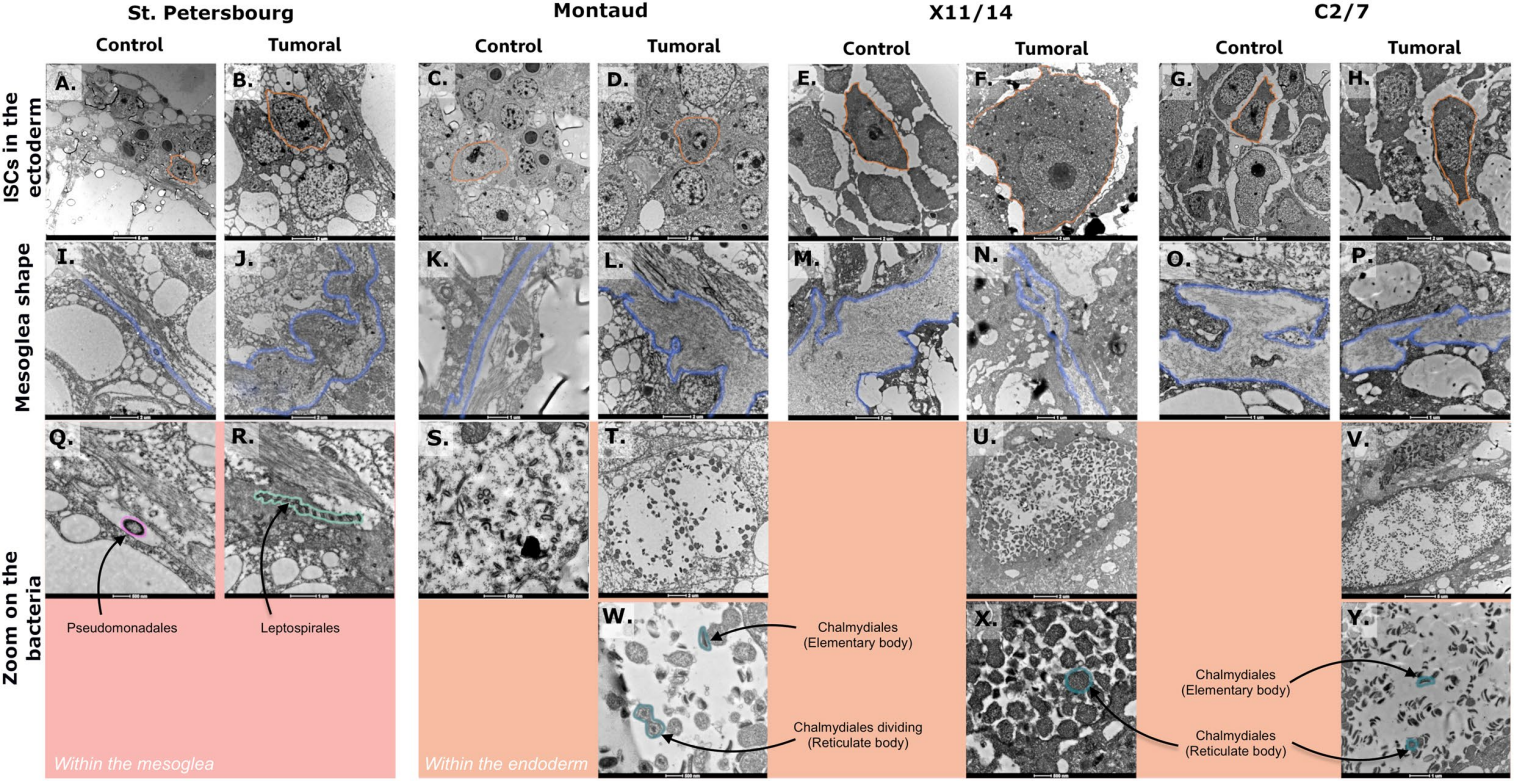
Differential localization of bacteria in tumor-bearing individuals. Transmission electronic pictures of the histological slides of control and tumorous individuals of each strain. (**A**–**H**) Pictures of the ectoderm zoom-in on the interstitial stem cells (ISC; scale bar from 2 to 5 µm). (**I**–**P**) Pictures of a portion of mesoglea (scale bar from 1 to 2 µm). Pictures zoom-in on visible bacteria (**Q**, **R**) in the mesoglea and (**S**–**Y**) in the endoderm (scale bar from 500 nm to 5 µm).

To better understand the localization of these bacteria in the tissues of control and tumorous hydras, we used transmission electronic microscopy (Fig. 4). First, for the St. Petersburg strain, we detected *Pseudomonas* in the mesoglea of control individuals (Fig. 4I,Q), while tumorous individuals show spirochetes (Fig. 4R) in their disturbed and enlarged mesoglea (Fig. 4J), consistent with earlier observations and with our 16S analysis. For the more recently sampled strains, the mesoglea (Fig. 4K–P) was empty of any visible bacteria and showed an irregular shape except for the control Montaud that harbor a filiform mesoglea (Fig. 4K). The endoderm of all the tumoral individuals of wild derived strains (Fig. 4T–V) contained an important number of vacuoles containing bacteria whose shape is typical of *Chlamydiales* (Fig. 4W,X,Y), consistent with the 16S rDNA analysis. Lastly, we detected a collection of bacteria of various shapes in the endoderm of control individuals from the Montaud strain (Fig. 4S), which is also consistent with the substantial fraction of unidentified bacteria in their microbiome.

## Discussion

Tumor development has been demonstrated in two female *Hydra* strains: one of *Hydra oligactis*, caused by the presence of specific host-associated bacteria (*Pseudomonas* and *Turneriella*) and another in *Pelmatohydra robusta* which is independent of any bacterial community^17,19^. In both cases, tumor development is the result of an accumulation of germline-restricted stem cells in the ectoderm, but no information existed on the generality of this phenomenon in other strains of *Hydra oligactis*. Here we demonstrate the occurrence of spontaneously appearing tumors in three lineages of *H. oligactis* from distinct populations from France and Hungary, sampled and raised in different labs, males and females. We describe and compare the histological traits associated with the tumorous phenotypes in these three hydra lineages and found that tumors show similarities across strains. Indeed, the presence of abnormal cells in the ectoderm of asexual individuals appears to be the material of all the tumors in each strain. However, we also demonstrate that tumors can be detected in a male strain as well, in addition to females. Furthermore, we detected differences in the composition of host-associated microbes between the strains. Specifically, we found intracellular bacteria from a non-described species of *Chlamydiales* (detected in most of our samples), that were forming visible large vacuoles only in tumor bearing individuals of all recently sampled strains. We discuss these findings in turn below.

The original description of tumors in hydras comes from a laboratory strain that has been maintained in the laboratory for 7 years before (*Hydra oligactis* St. Petersburg strain). Tumors in this strain are transmitted during asexual reproduction, resulting in lineages that express the phenotype with a high percentage (i.e., around 75% according to^24^). During routine maintenance of strains collected in France and Hungary, we detected sporadically appearing tumorous individuals that showed clear outgrowths on the body wall, a remarkable similarity with the morphology of the tumorous St. Petersburg individuals. Two of these strains (Montaud and X11/14) are females, while the third (C2/7) is a male, showing that tumor development is not limited to females in *Hydra oligactis*.

In the three recently isolated wild strains, as in the reference one, we detected differences in the histological structure between tumorous and non-tumorous individuals. Those results suggest that tumors in these strains are also composed of an increased number of abnormal ISCs (likely committed to differentiate into germline stem cells) in the ectoderm concomitantly with disruption of tissue organization. Indeed, the tissue thickness of both epithelia is increased and the size of ISCs present in the ectoderm is different. In the female strains, ISCs are significantly enlarged, while in the male strain, the area of those cells is reduced compared to the healthy individuals. These observations, particularly visible on the histological analyses, are consistent with the idea that the tumorous individuals contain an accumulation of abnormal germline stem cells in some areas of their ectoderm, since these cells have a larger area in females (being committed to egg or nurse cell development), and smaller area in males (being committed to sperm development)^25^. The variations in germline stem cell size in the tumors of the different females’ strains might also indicate that the accumulation has occurred at different steps of their differentiation, which is consistent with the literature^17^. To conclude, in all strains the tumors may have in common to be associated with the localized aggregation of abnormally differentiated germline stem cells, even under stable environmental conditions that are not supposed to trigger and allow gametogenesis in hydras. Our results strongly support that *Hydra oligactis* exhibit a susceptibility to germline tumor development, independently of their sex, rearing lab, or geographical origin. The increase in thickness of not only the ectoderm, but also the endoderm, certainly contributes significantly to the phenotype observed at the individual level. Although this increase in tissue layer thickness is commonly observed in our studies and in previous studies, the relationship between this increase and the presence of cheating ISCs remains to be understood. Other experiments, such as single cell transcriptomics, would allow a better understanding of the expression profile of each cell type and their respective contribution to the tumor phenotype. In addition, the maceration technique used in this study did not enable us to detect any significant difference in the number of ISCs at the individuals level, and thus the underlying mechanisms responsible for the local accumulation of abnormally differentiated ISCs remain unknown. Further investigation is needed to determine whether the presence of these “cheating cells” is due to the accumulation of differentiated cells escaping cell death, proliferation during advanced stages of gametogenesis, or both.

It is established that tumors, whether malignant or benign, can affect the fitness of the organisms that carry them in different ways^26^. The consequences of the previously described St. Petersburg lineage tumors are multiple and may vary depending on the lifespan, reproductive mode, or environment of the carrier individuals^17,19,24,27^. Thus, longitudinal follow-up of individuals carrying these sporadic tumors that we have described here would now be necessary to better understand their phenotypic consequences for the organism, as well as their proliferation dynamics during the life of the hydra. In addition, further research evaluating tumor prevalence among populations, under common or differentially controlled rearing conditions, might help us to better understand the potential triggers of tumoral proliferation in* Hydra oligactis.*

Tumor initiation and maintenance in the St. Petersburg strain are dependent on the presence of specific bacteria (*Pseudomonas* and *Leptospirales*) located in the mesoglea, while in the *P. robusta* strain the tumors were microbiome independent^19^. Compared to that, we characterized a different microbiome profile in the recent wild-derived strains and the already described ones. None of the recently sampled hydras (healthy or tumorous) seem to harbor bacteria in their mesoglea, Moreover, we detected the presence of the same bacteria identified (based on 16S fragment) as an undescribed species of *Chlamydiales*, as well as in some St. Petersburg individuals. Since the French and Hungarian laboratories have exchanged hydra strains in the past, we cannot exclude the hypothesis of a previous contamination with these Chlamydia strains before this study. Interestingly, there is no evidence of this bacterium in the natural microbiome of hydras sampled directly in the wild^28^, which can indicate either that these bacteria are rare or variably detectable. In addition, it is also possible that these bacteria became more abundant after few generations in the lab^21^.

Secondly, the TEM analysis revealed that those *Chlamydiales* were reproducing within vacuoles (i.e., inclusions) only visible in the endoderm of tumorous individuals. This same species of *Chlamydiales* was also detected in the non-tumorous polyps but without evidence of any visible inclusion, which might indicate a difference in the abundance, the activity, or the localization of this bacteria correlated to tumors presence. Further quantitative PCR analysis, as well as fluorescent hybridization in situ would be necessary to test these hypotheses. Whether the reproduction of the *Chlamydiales* is the cause or the consequence of the tumorigenesis also remain to be determined. Submitting these hydras to antibiotic treatments could in the future help us to understand if the *Chlamydiales* are required to initiate and/or maintain the tumors or if their presence is more opportunistic. In any case, this observation supports that hydra microbiome and the maintenance of tissue homeostasis are deeply interconnected^19,21,22,29^.

Although *Chlamydiales* are usually associated with human diseases (e.g. *C. trachomatis* and *C. pneumoniae*), there is a huge variety of hosts harboring different *Chlamydiales* species in the environment, which for the moment is still largely underestimated^30,31^. The phenotypic consequences associated with the presence of these bacteria are mostly unknown even if their virulence potential seems to be variable among the different families^32^. In addition, some *Chlamydiales* are even involved in long-term interactions with and defensive symbiosis in amoeba^33^. Thus, it does not seem so surprising to find as yet undocumented *Chlamydiales* in hydra and further study may even allow a better understanding of the diversity of the phenotypic impacts of these intracellular bacteria on animals.

Although we cannot unequivocally say that tumors in our strains are of germline stem cell origin and no other cell types are involved, the apparent local accumulation of stem cells in the tumor region, and the altered morphology of these cells (enlarged cell area in females, reduced cell area in males) relative to control animals is suggestive of a germline stem cell origin of tumors in all our strains. The reasons why germline stem cells appear to be particularly susceptible to tumor development in hydra, and why bacteria are sometimes associated with this phenomenon remain currently unclear. If hydra tumors were caused by somatic mutations accumulating in hydra cell lineages, then—all else being equal—germline stem cells should be less likely to develop somatic mutations compared to somatic cell lineages since they are slow cycling cells and the rate of cell proliferation strongly predicts the development of somatic mutations^25,34^. Furthermore, germline stem cells are thought to have substantially stronger DNA repair mechanisms compared to normal somatic tissues to reduce the mutational load of the offspring^35^. Therefore, germline stem cells should actually be less likely to develop tumors than other somatic cell types.

On the other hand, if tumors are the result of an interaction between hydra hosts and their bacterial community (as suggested by the bacterial origin of tumors in the St. Petersburg strain), then several scenarios might be envisaged^36^. First, the abnormal accumulation of germline stem cells might benefit the bacteria, if they can increase their fitness through this accumulation. For instance, if the bacteria causing tumor development are transferred vertically through eggs, then it might be in their interest to increase the sexual investment of the host. Although the presence of tumors in males seems to contradict this idea, recent evidence in Cnidarians indicates that components of the microbiome can be transmitted through sperm as well^37^. Secondly, the accumulation of germline stem cells in tumorous hydra might be an adaptive response from the side of the host to the presence of bacteria. For instance, female germline stem cells express the peptide *periculin*, which has potent antimicrobial effects^38^, and this could help to reduce microbial loads in heavily infected hydra. The presence of tumors in males is again enigmatic, since sperm precursors are not known to have a specifically high expression of antimicrobial molecule, studying existing expression datasets could probably bring us evidence. Alternatively, the increased number of germline stem cells in tumorous individuals could be an adaptive response from the side of the host to preserve its own fitness through terminal investment, i.e., increasing its reproductive effort in response to an increased mortality risk signaled by the presence of potentially pathogenic microorganisms. Future studies measuring the potential pathogenic effect of these bacteria are necessary to disentangle between these alternatives.

This comparative study lays the basis to further studies aiming to understand the context of the appearance of those tumors, which some have already proven their capacity to become transmissible^17,19^. Further research focusing on the ability of these sporadically occurring tumors cells to already possess, acquire through time, or remain unable to became transmissible may provide an unprecedented context for the study of transmissible cancers.

## Material and methods

### Hydra strains maintenance and monitoring

The Montaud strain was established from an individual collected in Montaud lake in France (43°44′52″N; 3°59′23″E) in April 2021 in the CREEC laboratory at Montpellier in August 2021. The control (i.e., healthy) and the tumoral Kiel's strains were obtained from Thomas Bosch’s laboratory and maintained in our lab for several months before the study. Individuals from the Montaud and Kiel lineages were maintained at 18 °C in Volvic© water, and fed three times per week according to the methodology described in Boutry and colleagues^14^. The X11/14 and C2/7 strains were provided by Jácint Tökölyi’s laboratory and originate from Hungary. The first individuals of X11/14 and C2/7 were sampled from Tiszadorogma, Hungary (47°67′12″N, 20°86′41″E) in August and September 2016 (see^39^ for a complete description of these strains). All these polyps were maintained at 18 °C, and fed 4 times per week according to the methodology described in^40^.

The sex of each lineage was determined by observing the development of gonads in a few clonal individuals, right after their sampling and after having reduced the rearing temperature between 8 to 10 °C. To detect swollen individuals that may appear sporadically in cultures, hydras were observed before each feeding. Individuals showing unusual morphology to the naked eye, reminiscent of tumorous individuals previously described in the literature, were then isolated and maintained separately before being included in any of the analyses presented here. Control individuals were randomly selected from the same batch that contained the swollen individuals.

### Bacterial metabarcoding analysis

We analyzed the bacterial microbiome through a 16S rDNA metabarcoding analysis of 6 to 12 individuals from the four strains with or without tumors, for a total of 70 individuals. We washed each individual four times in distilled sterile water and then froze them until analysis. The DNA was extracted with the DNeasy Blood & Tissue kit (QIAGEN©), following the manufacturer’s instructions with some adaptations to our biological model, including a 2-h lysis step and a final elution at a 30-µL volume. The amplification of the V4 variable region of the ribosomal 16S gene was performed as described in^41^. Controls were included at each extraction assay (4 assays in total): one controlling for kit’s reagents contamination and three controlling the bacteria present in hydra’s rearing water. PCR products were assigned individually with barcodes at the genomic platform (GenSeq, Montpellier University) that allows for the identification of 95 different PCR products onto the same MiSeq flow cell (Illumina). All PCR products were pooled, purified, and sequenced by the GenSeq platform using Illumina paired-end 2 × 300-bp technology with V2 chemistry. Procedures regarding to Illumina’s quality control, sequences clustering into OTUs, controls processing, and OTU assignation were performed using the pipeline FROGS^42^ implemented on a Galaxy workbench^43^ and following instructions described in^41^.

To compare bacterial composition between the different lineages and conditions of hydras in our study (tumoral and non-tumoral, for each lineage), we expressed OTUs’ representation using relative abundance values of OTU representing more than 50 reads in total. Relative abundance values were presented through a composition plot calculated using different FROGSTAT tools on Galaxy^43^ and modified with *ggplot2*^44^. The beta-diversity matrices (see in Supplementary) were constructed with a Jaccard distance method with Phyloseq^45^ in Rstudio (version 2022.02.1 + 461^46^).

### Morphological and histological analysis

Hydras used for histological slides were photographed using a binocular magnifier connected to OLYMPUS© EP50 camera and EPview software (v2.9.6_20201224; Olympus, Tokyo, Japan). The individuals were first immobilized using a 5% urethane solution and then immersed into a 2.5% Glutaraldehyde solution at 4 °C. Postfixation was achieved in FB-1% osmium tetraoxide for 1 h at 4 °C in the dark (same blocks were used for the TEM preparation). Excess fixing agent was eliminated during the dehydration step in a successive series of water solutions containing increasing amounts of acetone until reaching 100%. Finally, samples were embedded in epoxy resin (TAAB 812). Sections (80 nm thick) were stained with toluidine blue and observed, pictured, and measured under a microscope OLYMPUS©, the EP50 camera, and EPview software (v2.9.6_20201224; Olympus, Tokyo, Japan). The thickness measurements were obtained in triplicates based on three images of different locations on the same histological slides. For each of these triplicates, the thickness was calculated based on the average of three measures for the ectoderm and the endoderm and five measures for the mesoglea. In addition, the areas of two to three different ISCs were measured to obtain an average ISC area for the three different pictures selected on the slide. The thickness of the three tissue layers and the average area of the ISCs were compared by using general linear models, taking into account the effects of the strains and of the condition. The optimal model was selected using backwards model simplification followed by Likelihood Ratio Tests (see in Supplementary).

Samples for TEM were prepared as follows. Hydras were immersed in a solution of 2.5% glutaraldehyde in water overnight at 4 °C. They were then rinsed in water and post-fixed in a 0.5% osmic acid with 0.8% potassium Hexacyanoferrate trihydrate for 2 h in the dark at room temperature. Individuals were then rinsed twice in water and dehydrated in a gradual series of ethanol solutions (30–100%). The tissues were embedded in EmBed 812 using an Automated Microwave Tissue Processor from Electronic Microscopy, Leica EM AMW©. Thin sections of 1 µm were colored with toluidine Blue and observed with a light microscope. Ultra-thin Sects. (70 nm; Leica-Reichert Ultracut E) were collected at different levels of each block. These sections were counterstained with uranyl acetate 1.5% in 70% Ethanol and lead citrate and observed using a Tecnai F20 transmission electron microscope at 120 kV at the Institut des Neurosciences de Montpellier: Electronic Microscopy facilities.

### Maceration analysis

Five to six control and tumorous individuals from each strain (St Petersburg, Montaud, X11/14 and C2/7) were macerated following a protocol modified from^47^. Briefly, each hydra polyp was placed in a microtube in maceration solution (glycerin, glacial acetic acid, and water in a proportion of 1:1:13). The volume of the maceration solution was adjusted to the polyp size within the range of 10–30 µl to keep cell density roughly constant. Polyps were kept for 30 min in the maceration solution on room temperature and then dissociated by gently pipetting them up and down until no visible tissue fragments remained. For each individual, 3 replicate cell counting were made immediately after dissociation on an Euromex iScope microscope with phase contrast optics. For each replicate, around 50 epithelial cells were counted, as well as the interstitial cells found along with the epithelial cells. The proportions of interstitial stem cells in each strain were compared by using general linear models, taking into account the effects of the strains and of the condition. The optimal model was selected using backwards model simplification followed by Likelihood Ratio Tests (see in Supplementary).

## Supplementary Information

Supplementary Information.

## Data Availability

The datasets generated and/or analysed during the current study are available in the Mendeley Data Repository: 10.17632/453g8p489j.2. Microbiota 16S data are available in the Short Reads Archive under BioProject Accession No. PRJNA890148 (https://www.ncbi.nlm.nih.gov/bioproject/?term=PRJNA890148).

## References

[1] Aktipis, C. A. *The Cheating Cell*. (2020).

[2] Aktipis CA (2015). Cancer across the tree of life: cooperation and cheating in multicellularity. Philos. Trans. R. Soc. B Biol. Sci..

[3] Aktipis CA, Nesse RM (2013). Evolutionary foundations for cancer biology. Evol. Appl..

[4] Ujvari B, Roche B, Thomas F (2017). Ecology and Evolution of Cancer.

[5] Vittecoq M (2013). Cancer: a missing link in ecosystem functioning?. Trends Ecol. Evol..

[6] Cairns J (1975). Mutation selection and the natural history of cancer. Nature.

[7] Nowell PC (1976). The clonal evolution of tumor cell populations. Science.

[8] Maley CC (2017). Classifying the evolutionary and ecological features of neoplasms. Nat. Rev. Cancer.

[9] Merlo LMF, Pepper JW, Reid BJ, Maley CC (2006). Cancer as an evolutionary and ecological process. Nat. Rev. Cancer.

[10] Domazet-Lošo T, Tautz D (2010). Phylostratigraphic tracking of cancer genes suggests a link to the emergence of multicellularity in metazoa. BMC Biol..

[11] Giraudeau M, Sepp T, Ujvari B, Ewald PW, Thomas F (2018). Human activities might influence oncogenic processes in wild animal populations. Nat. Ecol. Evol..

[12] Madsen T, Ujvari B, Roche B, Thomas F (2017). Cancer prevalence and etiology in wild and captive animals. Ecology and Evolution of Cancer.

[13] Hamede R (2020). The ecology and evolution of wildlife cancers: Applications for management and conservation. Evol. Appl..

[14] Boutry J (2022). Tumors (re)shape biotic interactions within ecosystems: Experimental evidence from the freshwater cnidarian *Hydra*. Sci. Total Environ..

[15] Reiter S, Crescenzi M, Galliot B, Buzgariu W (2012). *Hydra*, a versatile model to study the homeostatic and developmental functions of cell death. Int. J. Dev. Biol..

[16] Tomczyk S, Fischer K, Austad S, Galliot B (2015). *Hydra*, a powerful model for aging studies. Invertebr. Reprod. Dev..

[17] Domazet-Lošo T (2014). Naturally occurring tumours in the basal metazoan *Hydra*. Nat. Commun..

[18] Ujvari B, Gatenby RA, Thomas F (2016). The evolutionary ecology of transmissible cancers. Infect. Genet. Evol..

[19] Rathje K (2020). Dynamic interactions within the host-associated microbiota cause tumor formation in the basal metazoan *Hydra*. PLoS Pathog..

[20] Franzenburg S (2013). Distinct antimicrobial peptide expression determines host species-specific bacterial associations. Proc. Natl. Acad. Sci. U.S.A.

[21] Fraune S, Bosch TCG (2007). Long-term maintenance of species-specific bacterial microbiota in the basal metazoan Hydra. Proc. Natl. Acad. Sci. U.S.A.

[22] Lange, C. Molecular analysis of the innate immune response in *Hydra*. (2010).

[23] Bennett GM, Moran NA (2015). Heritable symbiosis: The advantages and perils of an evolutionary rabbit hole. Proc. Natl. Acad. Sci..

[24] Boutry J (2022). Tumors alter life history traits in the freshwater cnidarian, *Hydra oligactis*. iScience.

[25] Nishimiya-Fujisawa C, Kobayashi S, Kobayashi K, Kitano T, Iwao Y, Kondo M (2018). Roles of germline stem cells and somatic multipotent stem cells in hydra sexual reproduction. Reproductive and Developmental Strategies: The Continuity of Life.

[26] Boutry J (2022). The evolution and ecology of benign tumors. Biochim. Biophys. Acta (BBA) Rev. Cancer.

[27] Tissot, S. *et al.* Cancer’s vulnerability to food availability is evolutionarily conserved: diet modulates tumorigenesis in both Hydra and zebrafish. (submitted).

[28] Taubenheim J, Miklós M, Tökölyi J, Fraune S (2022). Population differences and host species predict variation in the diversity of host-associated microbes in hydra. Front. Microbiol..

[29] Fraune S, Abe Y, Bosch TCG (2009). Disturbing epithelial homeostasis in the metazoan Hydra leads to drastic changes in associated microbiota. Environ. Microbiol..

[30] Corsaro D, Valassina M, Venditti D (2003). Increasing diversity within *Chlamydiae*. Crit. Rev. Microbiol..

[31] Horn M (2008). *Chlamydiae* as symbionts in eukaryotes. Annu. Rev. Microbiol..

[32] Collingro A (2011). Unity in variety–the pan-genome of the *Chlamydiae*. Mol. Biol. Evol..

[33] König L (2019). Symbiont-mediated defense against *Legionella pneumophila* in amoebae. mBio.

[34] Tomasetti C, Vogelstein B (2015). Variation in cancer risk among tissues can be explained by the number of stem cell divisions. Science.

[35] Maklakov AA, Immler S (2016). The expensive germline and the evolution of ageing. Curr. Biol..

[36] Ewald PW (1980). Evolutionary biology and the treatment of signs and symptoms of infectious disease. J. Theor. Biol..

[37] Baldassarre L (2021). Contribution of maternal and paternal transmission to bacterial colonization in *Nematostella vectensis*. Front. Microbiol..

[38] Fraune S (2010). In an early branching metazoan, bacterial colonization of the embryo is controlled by maternal antimicrobial peptides. Proc. Natl. Acad. Sci. U.S.A.

[39] Sebestyén F, Miklós M, Iván K, Tökölyi J (2020). Age-dependent plasticity in reproductive investment, regeneration capacity and survival in a partially clonal animal (*Hydra oligactis*). J. Anim. Ecol..

[40] Tökölyi J (2016). Effects of food availability on asexual reproduction and stress tolerance along the fast–slow life history continuum in freshwater hydra (Cnidaria: Hydrozoa). Hydrobiologia.

[41] Binetruy F, Dupraz M, Buysse M, Duron O (2019). Surface sterilization methods impact measures of internal microbial diversity in ticks. Parasit. Vectors.

[42] Escudié F (2018). FROGS: Find, rapidly, OTUs with galaxy solution. Bioinformatics.

[43] Goecks J, Nekrutenko A, Taylor J, The Galaxy Team (2010). Galaxy: A comprehensive approach for supporting accessible, reproducible, and transparent computational research in the life sciences. Genome Biol..

[44] Wickham H (2016). ggplot2: Elegant Graphics for Data Analysis.

[45] McMurdie PJ, Holmes S (2013). phyloseq: an R package for reproducible interactive analysis and graphics of microbiome census data. PLoS ONE.

[46] RStudio Team. RStudio: Integrated Development Environment for R. (2022).

[47] David CN (1973). A quantitative method for maceration of hydra tissue. Wilhelm Roux Arch. Entwickl. Mech. Org..

