# Supplementary material for: Spontaneously occurring tumors in different wild-derived strains of hydra
Source: Sci Rep. 2023 May 8;13:7449. doi: 10.1038/s41598-023-34656-0 (PMC10167321; doi:10.1038/s41598-023-34656-0)
Supplement: Supplementary file 1 — Supplementary Information. [file 41598_2023_34656_MOESM1_ESM.pdf]

Supplementary tables: model outputs

Supplementary Table 1. Coefficients tables from linear models (ANOVA) to explain the ectoderm thickness in tumorous vs. non-tumorous (Status) hydra from different strains (Strain). The table shows parameter estimates and their standard errors (in brackets). Significance levels are shown by asterisks.

	Strain*Status	Strain+Status	Strain	Status
StrainMontaud	1.567*	1.480**	1.480	
	(0.570)	(0.434)	(1.118)	
StrainX11/14	1.737**	1.107*	1.107	
	(0.570)	(0.434)	(1.118)	
StrainC2/7	2.447***	1.627**	1.627	
	(0.570)	(0.434)	(1.118)	
StatusTum	4.040***	3.272***		3.272***
	(0.570)	(0.307)		(0.394)
StrainMontaud × StatusTum	-0.173			
	(0.806)			
StrainX11/14 × StatusTum	-1.260			
	(0.806)			
StrainC2/7 × StatusTum	-1.640+			
	(0.806)			
Num.Obs.	24	24	24	24
R2	0.908	0.873	0.115	0.758
R2 Adj.	0.868	0.847	-0.018	0.747
AIC	59.1	60.8	105.4	70.3
BIC	69.7	67.8	111.3	73.8
Log.Lik.	-20.565	-24.380	-47.718	-32.137
RMSE	0.57	0.67	1.77	0.92

+ $p < 0.1$, * $p < 0.05$, ** $p < 0.01$, *** $p < 0.001$

Supplementary Table 2. Coefficients tables from linear models (ANOVA) to explain mesoglea thickness in tumorous vs. non-tumorous (Status) hydra from different strains (Strain). The table shows parameter estimates and their standard errors (in brackets). Significance levels are shown by asterisks.

	Strain*Status	Strain+Status	Strain	Status
StrainMontaud	0.193	0.525+	0.525+	
	(0.355)	(0.262)	(0.271)	
StrainX11/14	0.490	0.377	0.377	
	(0.355)	(0.262)	(0.271)	
StrainC2/7	0.587	0.422	0.422	
	(0.355)	(0.262)	(0.271)	
StatusTum	0.257	0.283		0.283
	(0.355)	(0.185)		(0.192)
StrainMontaud × StatusTum	0.663			
	(0.502)			
StrainX11/14 × StatusTum	-0.227			
	(0.502)			
StrainC2/7 × StatusTum	-0.330			
	(0.502)			
Num.Obs.	24	24	24	24
R2	0.434	0.267	0.177	0.090
R2 Adj.	0.187	0.112	0.053	0.049
AIC	36.4	36.6	37.4	35.8
BIC	47.0	43.7	43.3	39.3
Log.Lik.	-9.198	-12.313	-13.703	-14.904
RMSE	0.35	0.40	0.43	0.45

+ $p < 0.1$, * $p < 0.05$, ** $p < 0.01$, *** $p < 0.001$

Supplementary Table 3. Coefficients tables from linear models (ANOVA) to explain endoderm thickness in tumorous vs. non-tumorous (Status) hydra from different strains (Strain). The table shows parameter estimates and their standard errors (in brackets). Significance levels are shown by asterisks.

	Strain*Status	Strain+Status	Strain	Status
StrainMontaud	1.970	0.848	0.848	
	(1.336)	(1.278)	(2.191)	
StrainX11_14	9.747***	6.033***	6.033*	
	(1.336)	(1.278)	(2.191)	
StrainC2_7	4.813**	4.425**	4.425+	
	(1.336)	(1.278)	(2.191)	
StatusTum	8.310***	5.698***		5.698***
	(1.336)	(0.904)		(1.353)
StrainMontaud × StatusTum	-2.243			
	(1.889)			
StrainX11_14 × StatusTum	-7.427**			
	(1.889)			
StrainC2_7 × StatusTum	-0.777			
	(1.889)			
Num.Obs.	24	24	24	24
R2	0.902	0.787	0.340	0.446
R2 Adj.	0.859	0.742	0.241	0.421
AIC	100.0	112.6	137.7	129.5
BIC	110.6	119.7	143.6	133.1
Log.Lik.	-41.008	-50.322	-63.870	-61.763
RMSE	1.34	1.97	3.46	3.17

+ $p < 0.1$, * $p < 0.05$, ** $p < 0.01$, *** $p < 0.001$

Supplementary Table 4. Coefficients tables from linear models (ANOVA) to explain interstitial cell area in tumorous vs. non-tumorous (Status) hydra from different strains (Strain). The table shows parameter estimates and their standard errors (in brackets). Significance levels are shown by asterisks.

	Strain*Status	Strain+Status	Strain	Status
StrainMontaud	0.312	-0.387	-0.317	
	(0.725)	(0.568)	(0.599)	
StrainX11/14	0.350	0.377	0.377	
	(0.649)	(0.540)	(0.571)	
StrainC2/7	0.527	-0.687	-0.687	
	(0.649)	(0.540)	(0.571)	
StatusTum	1.607*	0.705+		0.686
	(0.649)	(0.392)		(0.403)
StrainMontaud × StatusTum	-1.315			
	(0.973)			
StrainX11/14 × StatusTum	0.053			
	(0.917)			
StrainC2/7 × StatusTum	-2.427*			
	(0.917)			
Num.Obs.	23	23	23	23
R2	0.574	0.292	0.165	0.121
R2 Adj.	0.376	0.135	0.033	0.080
AIC	62.9	68.6	70.4	67.5
BIC	73.1	75.4	76.0	70.9
Log.Lik.	-22.429	-28.281	-30.184	-30.767
RMSE	0.64	0.83	0.90	0.92

+ p < 0.1, * p < 0.05, ** p < 0.01, *** p < 0.001

Supplementary Table 5. Coefficients tables from linear models (ANOVA) to explain the number of interstitial cells in tumorous vs. non-tumorous (Status) hydra from different strains (Strain). The table shows parameter estimates and their standard errors (in brackets). Significance levels are shown by asterisks.

	Strain*Status	Strain+Status	Strain	Status
StrainMontaud	-0.046	-0.038	-0.038	
	(0.052)	(0.037)	(0.038)	
StrainX11/14	-0.041	0.021	0.021	
	(0.054)	(0.039)	(0.039)	
StrainC2/7	-0.031	0.025	0.025	
	(0.054)	(0.039)	(0.039)	
StatusTum	-0.030	0.034		0.034
	(0.056)	(0.026)		(0.026)
StrainMontaud × StatusTum	0.017			
	(0.074)			
StrainX11/14 × StatusTum	0.124			
	(0.076)			
StrainC2/7 × StatusTum	0.112			
	(0.076)			
Num.Obs.	48	48	48	48
R2	0.207	0.118	0.083	0.034
R2 Adj.	0.068	0.035	0.021	0.013
AIC	-86.9	-87.8	-88.0	-89.5
BIC	-70.1	-76.6	-78.6	-83.8
Log.Lik.	52.466	49.893	48.980	47.729
RMSE	0.08	0.09	0.09	0.09

+ p < 0.1, * p < 0.05, ** p < 0.01, *** p < 0.001

Supplementary figures

Supplementary Figure 1. Histological slides showing St. Petersburg normal and tumorous animals. Longitudinal cross-sections are shown above, while close-ups of the ectoderm and endoderm are shown below.

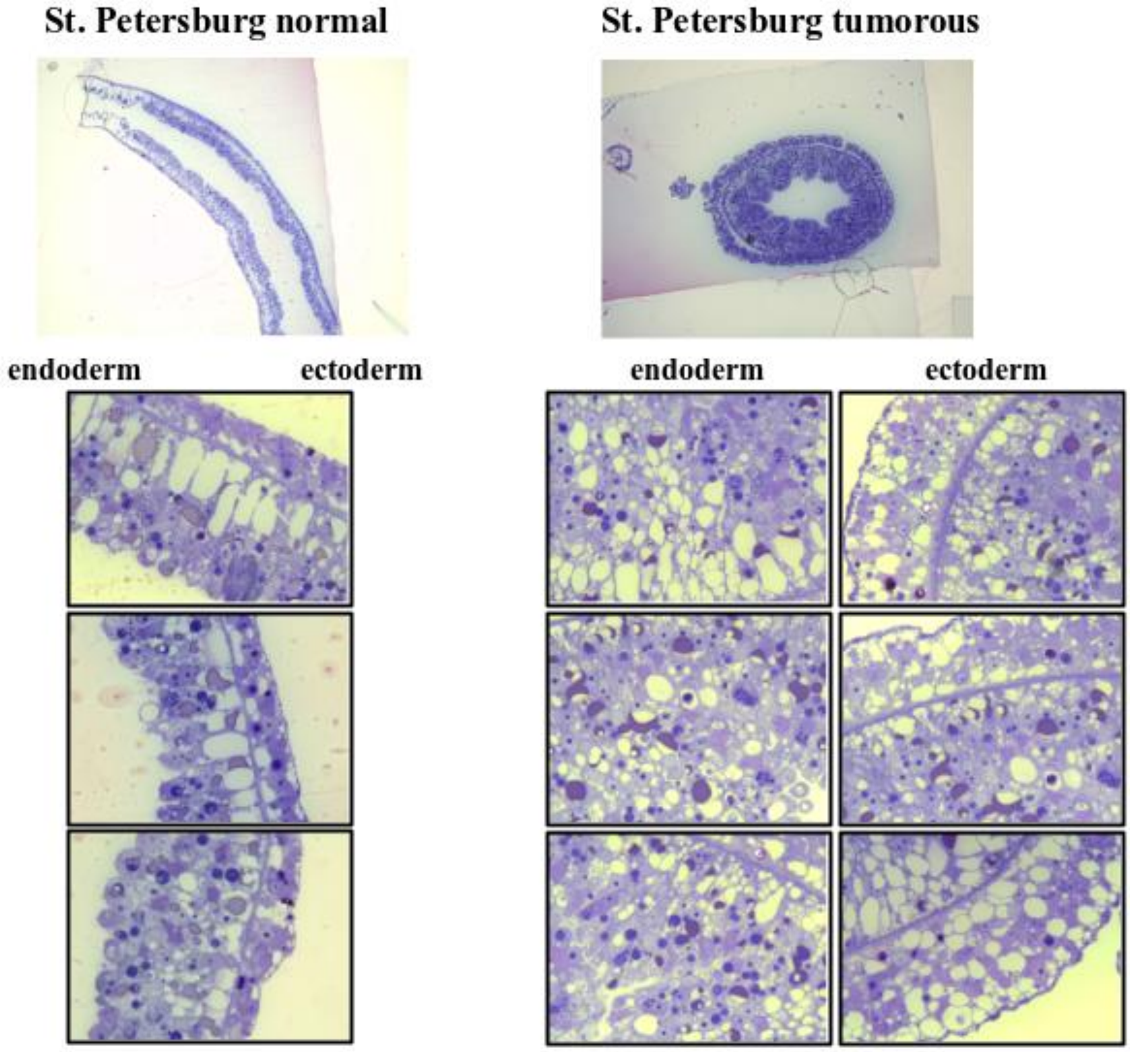

Supplementary Figure 2. Histological slides showing Montaud normal and tumorous animals. Longitudinal cross-sections are shown above, while close-ups of the ectoderm and endoderm are shown below.

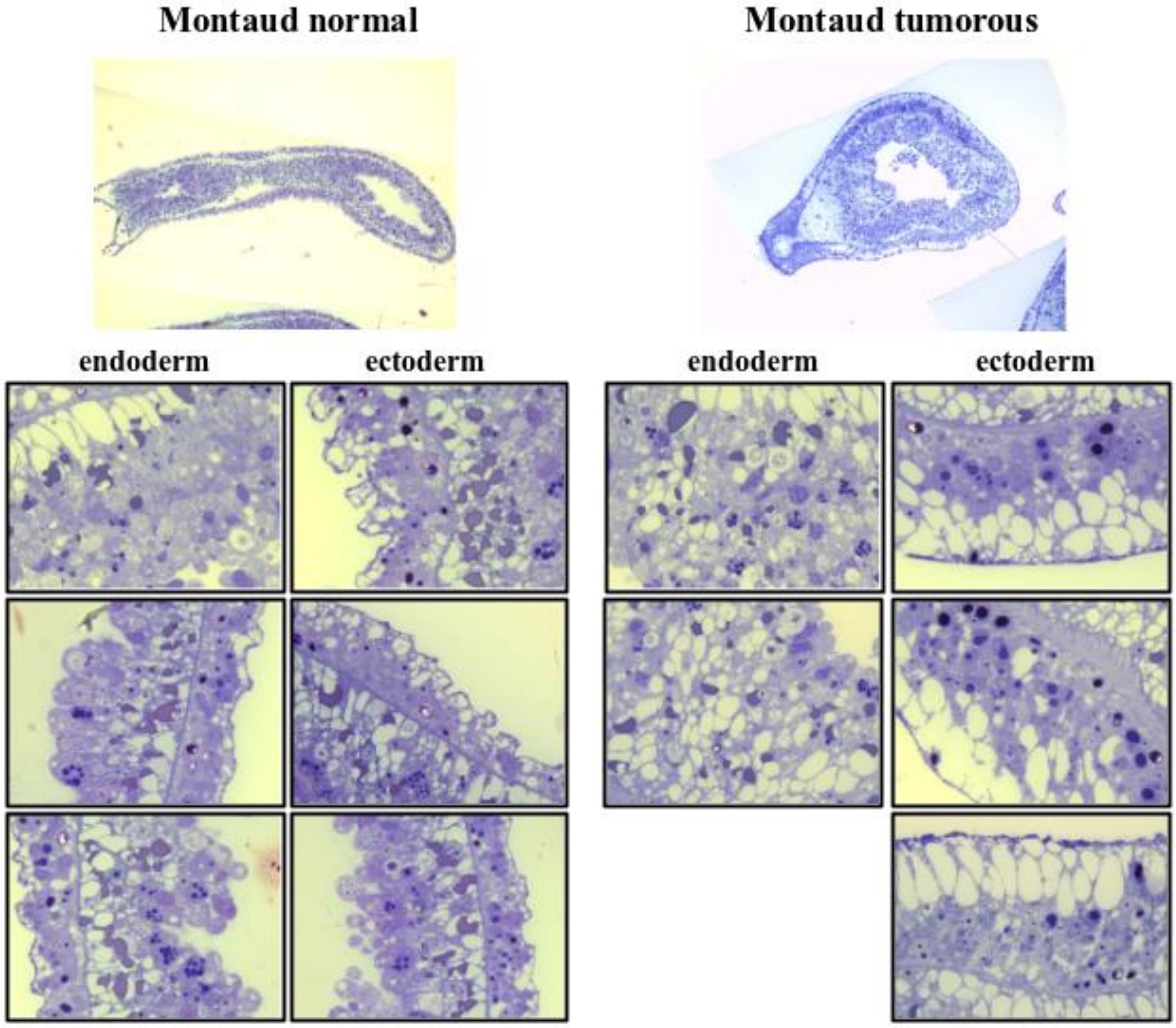

Supplementary Figure 3. Histological slides showing X11/14 normal and tumorous animals. Longitudinal cross-sections are shown above, while close-ups of the ectoderm and endoderm are shown below.

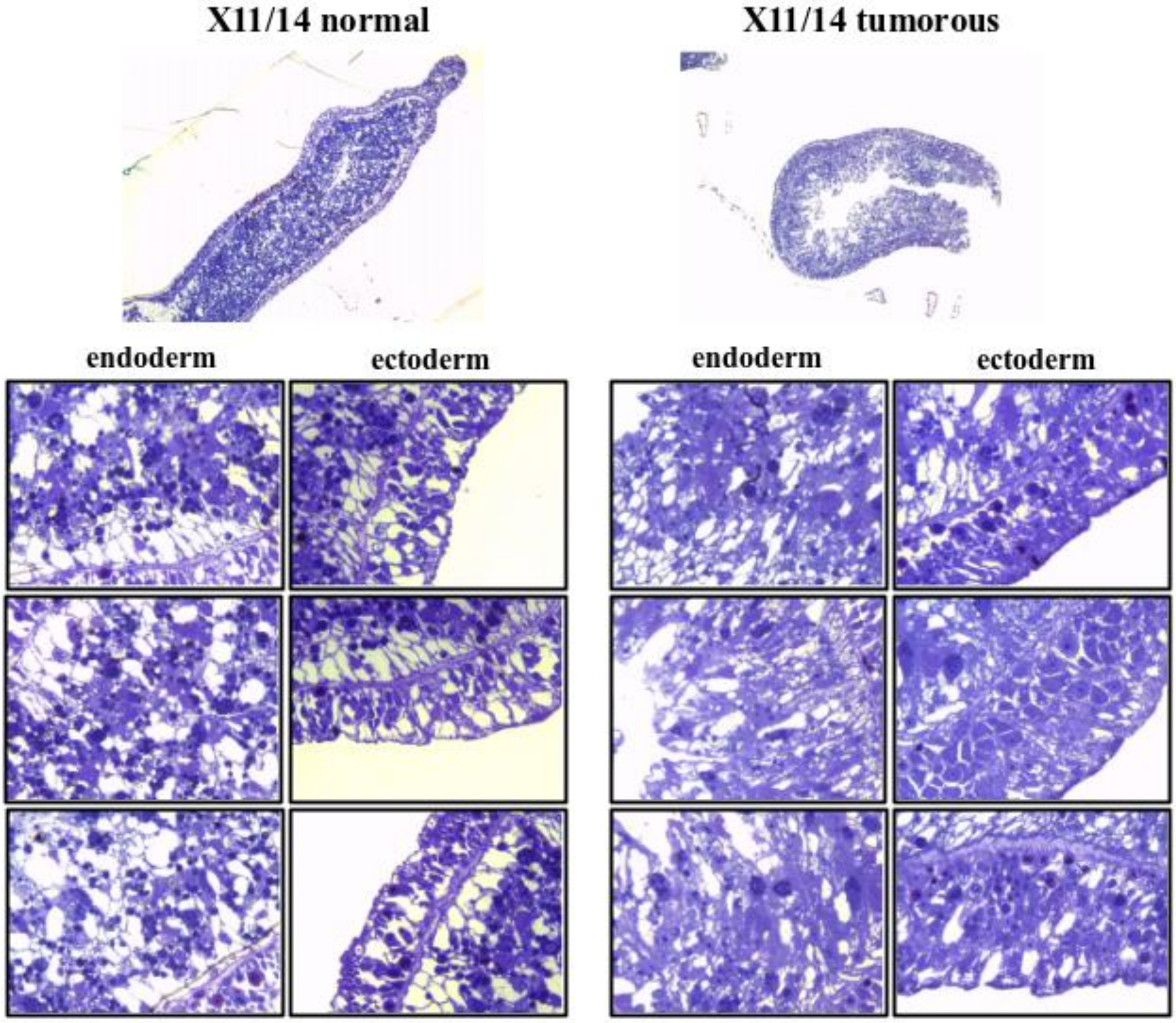

Supplementary Figure 4. Histological slides showing C2/7 normal and tumorous animals. Longitudinal cross-sections are shown above, while close-ups of the ectoderm and endoderm are shown below.

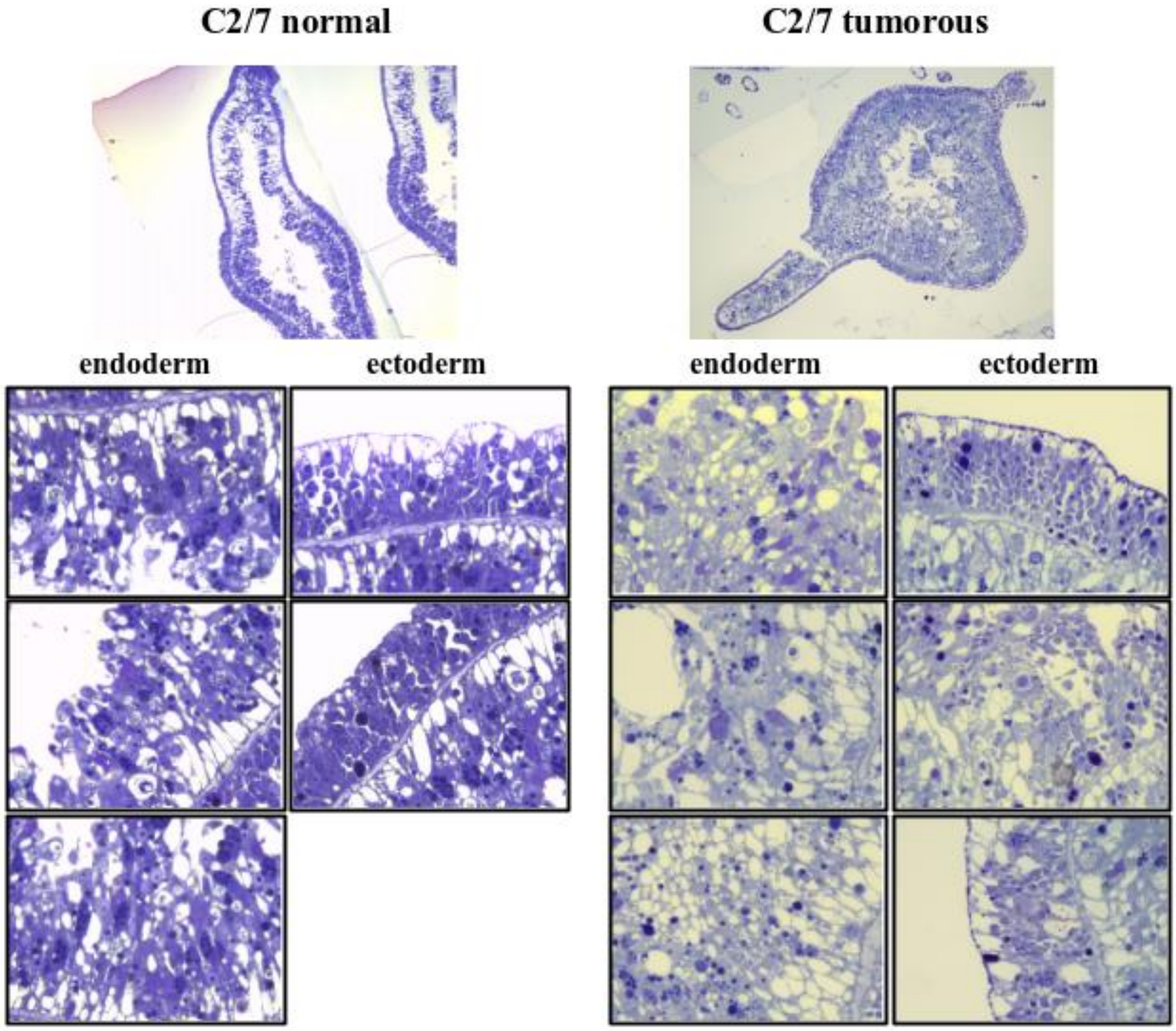

Supplementary Figure 5: Microbiome beta diversity ordination plot
Distances were calculated using Jaccard method and represented by MDS (multidimensional scaling). Each color represents a group of hydras from the same lineage and status (T: tumorous, NT: Non-tumorous). Ellipses represents the lineages of each group, independently of their status.

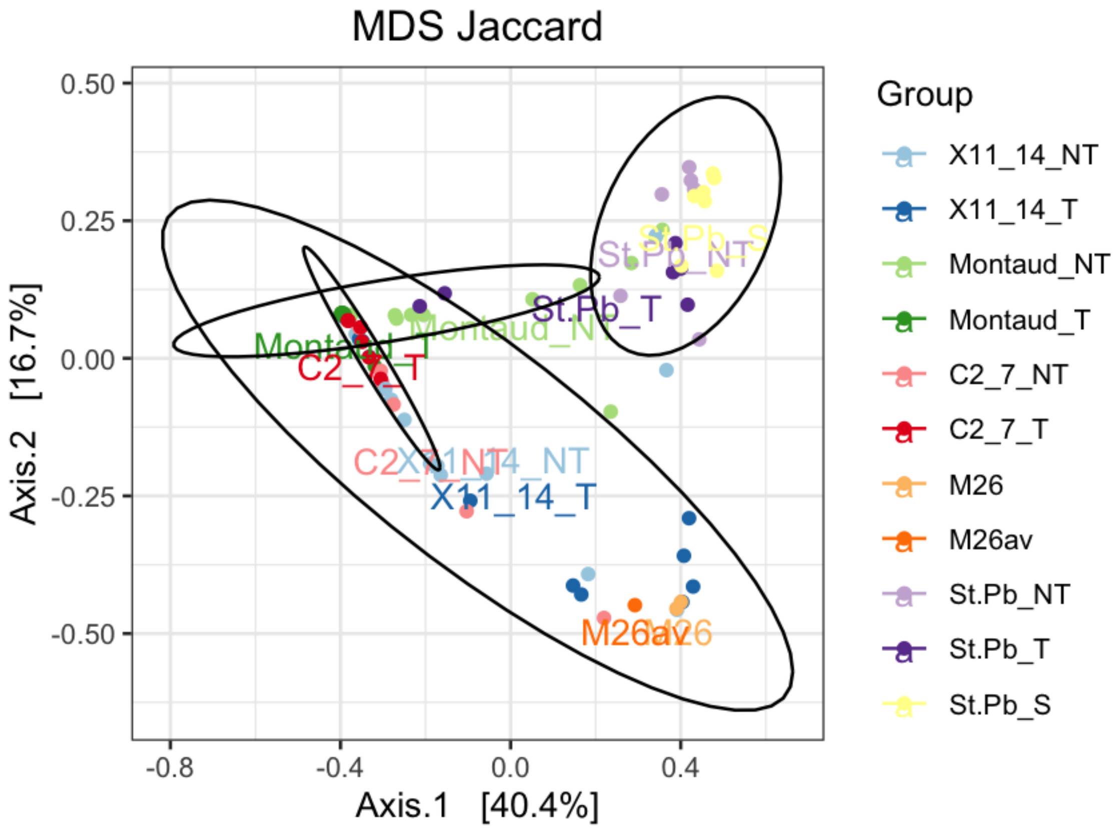

N.B: M26 lineage (in orange) was not considered in the main analysis given the very small sample size. This beta-diversity also include a group of St. Petersbourg hydras that were sick (St. Pb_S, in yellow), which were included here only on an exploratory purpose.
